# Deep Learning-Based Automatic Modulation Classification for OFDM Signals: From Synthetic Training to OTA Evaluation

**DOI:** 10.3390/s26102945

**Published:** 2026-05-08

**Authors:** Raluca Nelega, Mate-Marton Mezei, Zsolt Alfred Polgar, Gergo Kovacs, Emanuel Puschita

**Affiliations:** 1 National Institute for Research and Development of Isotopic and Molecular Technologies, 67-103 Donat Street, 400293 Cluj-Napoca, Romania; raluca.nelega@itim-cj.ro (R.N.); gergo.kovacs@itim-cj.ro (G.K.); 2 Communications Department, Technical University of Cluj-Napoca, 26-28 George Baritiu Street, 400027 Cluj-Napoca, Romania; mate.mezei@com.utcluj.ro (M.-M.M.); zsolt.polgar@com.utcluj.ro (Z.A.P.)

**Keywords:** automatic modulation classification, CNN, cross-domain generalization, deep learning, signal processing

## Abstract

To address the growing congestion of the radio frequency (RF) spectrum, Cognitive Radio (CR) systems employ Automatic Modulation Classification (AMC) to dynamically optimize spectrum utilization without introducing protocol overhead. In modern Orthogonal Frequency Division Multiplexing (OFDM) standards, effective AMC requires advanced signal-processing techniques capable of accurately identifying modulation schemes under dynamic channel conditions. Therefore, maintaining robust performance under realistic environments remains a fundamental challenge. This paper evaluates how dataset scale, synthetic impairments, and hardware-induced signal impairments affect the cross-domain generalization of a Convolutional Neural Network (CNN) architecture for OFDM Automatic Modulation Classification (AMC), using 2D amplitude-phase histograms for signal representation. To assess these effects, the CNN is trained on five distinct datasets, encompassing both synthetically generated signals with varying scales and synchronization impairments, as well as a conducted hardware dataset. The cross-domain generalization of the trained models is assessed by evaluating them on a completely unseen indoor Over-The-Air (OTA) dataset collected across 13 distinct positions. Statistical analysis demonstrates that the large-scale synchronization-impaired synthetic dataset achieves the best generalization performance, reaching a mean indoor OTA accuracy of 93.36% and outperforming the limited-size conducted hardware dataset. Overall, this study demonstrates the critical role of data-generation strategies and establishes a robust baseline for achieving reliable cross-domain generalization of CNN-based AMC.

## 1. Introduction

The rapid expansion of wireless communication systems and the proliferation of Internet of Things (IoT) devices have led to unprecedented congestion within the radio frequency (RF) spectrum [1]. Efficient management of this finite resource has therefore become a critical challenge, driving the development of intelligent networking paradigms such as Cognitive Radio (CR). Leveraging the flexibility of Software-Defined Radio (SDR) architectures [2], a CR system continuously senses its surrounding RF environment and dynamically adapts its transmission parameters to optimize spectrum utilization while minimizing interference [3]. A fundamental prerequisite for this adaptability is Automatic Modulation Classification (AMC)—the process of identifying the modulation scheme of an intercepted signal in the presence of noise, fading, and interference, without prior knowledge of the transmitter’s parameters. While modern transmitters can explicitly signal their modulation type within each frame to manage data rates and bandwidth, this creates significant protocol overhead that degrades spectrum efficiency. AMC eliminates this overhead by allowing the receiver to autonomously detect the modulation type, ensuring accurate data recovery while maximizing available bandwidth [4].

In modern wireless communication standards (i.e., Wi-Fi 6 and 5G), Orthogonal Frequency Division Multiplexing (OFDM) has become a dominant transmission technique due to its high spectral efficiency, strong resilience to multipath fading, and flexible bandwidth allocation capabilities. As stated in [5], AMC in OFDM-based systems requires more advanced processing than the direct use of raw time-domain samples, since each sample represents only a limited fraction of the information contained in a complete OFDM symbol. Consequently, effective AMC approaches must be capable of accurately identifying modulation schemes in OFDM transmissions, particularly under dynamic channel conditions and in the presence of noise.

However, cross-domain generalization remains a key challenge in this setting. Models trained and evaluated under controlled or simulated conditions often degrade when exposed to OTA environments with varying channel conditions, hardware impairments, and interference patterns.

To address this challenge, this paper evaluates the impact of dataset scale, synthetic impairments, and hardware-induced signal impairments on the cross-domain generalization of a CNN for OFDM AMC using a 2D amplitude-phase histogram signal representation.

To ensure methodological rigor, the end-to-end processing chain of this study is structured into four distinct stages: (1) signal generation, (2) feature extraction, (3) model training, and (4) cross-domain evaluation.

In the first stage (signal generation), baseband in-phase and quadrature (I/Q) signals are synthetically generated, incorporating multipath fading and noise effects. These baseline signals are then either digitally augmented to include simulated synchronization impairments or physically transmitted through conducted SDR setups, resulting in five distinct training datasets: (D1) a mid-scale perfectly synchronized synthetic dataset, (D2) a mid-scale synchronization-impaired synthetic dataset, (D3) a large-scale synchronization-impaired synthetic dataset, (D4) a small-scale synchronization-impaired synthetic dataset, and (D5) a small-scale conducted hardware dataset.

In the second stage (feature extraction), the raw signals are processed through a standardized pipeline, where they are converted into 50 × 50 amplitude-phase histograms and normalized on a per-sample basis to ensure consistent energy scaling across domains.

In the third stage (model training), the resulting normalized 2D histograms are used to train a CNN architecture, with each of the five dataset scenarios trained over ten independent runs to ensure statistical reliability.

In the fourth stage (cross-domain evaluation), the trained models are evaluated on an unseen, real-world OTA dataset collected across 13 indoor testing positions, enabling a systematic comparison of how different training datasets influence cross-domain generalization performance.

The main **contributions** of this paper are:

- **Evaluation of training dataset scale and signal impairments:** The effects of dataset scale and signal impairments are evaluated by comparing CNN models trained on perfectly synchronized synthetic data, synchronization-impaired synthetic data, and real hardware measurements. This analysis examines how dataset size, simulation assumptions, synthetic impairments, and hardware-induced signal impairments influence classification performance on OTA data.
- **Cross-domain generalization analysis:** To validate practical robustness, the trained models are evaluated on a completely unseen OTA dataset collected across 13 distinct indoor positions, enabling a systematic analysis of their ability to generalize across new spatial environments and channel conditions.
- **Multi-tier dataset generation framework:** A structured data-generation framework is introduced to benchmark training strategies, spanning perfectly synchronized synthetic data, synchronization-impaired synthetic data, and conducted hardware measurements. The generated datasets are publicly available online at [6].

The **remainder** of this paper is organized as follows: Section 2 reviews state-of-the-art techniques for Automatic Modulation Classification and their training strategies. Section 3 describes the dataset-generation process, and Section 4 details the proposed CNN architecture along with the training hyperparameters. Section 5 presents the experimental results, and Section 6 concludes the paper.

## 2. State of the Art

Given its fundamental role in enabling intelligent spectrum awareness, AMC has attracted significant research attention within the literature. Over the years, a wide range of techniques have been proposed, spanning conventional feature-based approaches and, more recently, data-driven deep learning methods. Accordingly, studies such as [7,8] provide comprehensive surveys of traditional as well as deep learning-based AMC techniques reported in the literature.

In ref. [7], AMC methods are classified into three main categories: (1) decision-theoretic, (2) feature-based (FB) and (3) deep learning (DL). However, as highlighted in ref. [7], traditional decision-theoretic and feature-based (FB) methods face significant challenges when applied to Orthogonal Frequency Division Multiplexing (OFDM) signals. Because decision-theoretic methods rely on statistical characteristics of the received signals, their high computational complexity and sensitivity to unknown channel parameters make them impractical for dynamic, real-world environments. Similarly, FB methods, such as those employing higher-order cumulants [9] and cyclostationarity [10], are computationally complex, which significantly reduces their overall efficiency [11]. Moreover, they depend heavily on manual expert feature extraction, which is particularly difficult for OFDM signals, as the orthogonality between OFDM subcarriers is easily degraded by frequency offsets and phase noise, leading to severe inter-subcarrier interference [8].

With the advancements in the field of artificial intelligence, recent research increasingly adopts deep learning (DL) approaches for AMC. However, a critical bottleneck in practical AMC for OFDM systems remains achieving accurate symbol synchronization at the input of the classifier. In real-world, non-cooperative spectrum sensing, a receiver rarely has perfect timing or frequency alignment with the transmitter. Yet, many AMC methods do not adequately address synchronization impairments. For instance, the authors in [11,12] bypass the synchronization problem entirely by feeding ideal, pre-segmented I/Q samples directly into the network, implicitly assuming that perfect symbol timing and carrier recovery have already been achieved prior to classification. Other approaches acknowledge the issue, but treat it as a ‘black-box’. In ref. [13], a 1D CNN with residual connections is applied directly to raw I/Q samples, while ref. [14] proposes a complex bi-stream CNN-LSTM with attention mechanisms. Rather than explicitly modeling and compensating for these impairments, these methods rely on network over-parameterization to absorb synchronization errors. This strategy often results in limited interpretability and poor generalization under dynamic channel conditions.

To overcome the reliance on black-box over-parameterization, the authors in ref. [5] perform AMC by feeding two-dimensional (2D) histogram representations of OFDM signals to a CNN. Instead of directly processing raw time-domain I/Q samples, the method first extracts a feature sequence from the normalized amplitude and phase differences between consecutive OFDM symbols. The statistical distribution of these features is then represented as a 2D histogram, where the horizontal axis corresponds to normalized amplitude, and the vertical axis represents phase difference. The authors evaluated and validated this approach on Wi-Fi 6 (using High Throughput (HT) and High Efficiency (HE) formats) and 5G downlink signals. The resulting 2D histogram representation offers several key advantages:

- **Robustness to symbol-level synchronization errors:** By relying on phase differences between adjacent symbols, identical phase drifts mathematically cancel out.
- **Invariance to sequence length:** The fixed dimensions of the histogram allow the network to process signals of any arbitrary duration.
- **Robustness to carrier frequency offset (CFO):** CFO estimation errors simply manifest as a uniform vertical shift along the histogram’s phase axis.
- **Broad IEEE 802.11 applicability:** By exploiting basic OFDM properties rather than protocol-specific preambles, the method natively supports non-HT, HT, VHT, and HE IEEE 802.11 transmission formats.

Moreover, the authors in ref. [5] evaluate their model using two isolated datasets: (1) synthetic signals degraded exclusively by Additive White Gaussian Noise (AWGN), and (2) measured OTA data. However, the classifier is strictly trained and tested within the same data domain. By omitting cross-scenario evaluations such as testing the synthetically trained model on OTA data, the study does not address how well the model generalizes to unseen environments.

Training and evaluating AMC deep learning models on the same dataset remains a common practice in the literature [15,16,17]. However, recent studies indicate that the absence of evaluation on independent datasets can mask significant performance limitations, as models trained on isolated data often experience substantial degradation when applied to real-world wireless environments [18,19]. One key reason for this limitation is the difficulty of collecting large-scale, accurately labelled hardware datasets. As a result, researchers face an inherent trade-off between the scalability and convenience of synthetically generated data and the physical realism provided by smaller hardware-derived measurements [19].

To address these limitations, the present study evaluates the generalization capability of a CNN architecture for AMC using the same signal representation as proposed in ref. [5]. Unlike prior work confined to a single data domain, this study benchmarks the CNN across distinct training datasets to identify the most effective training strategy. Moreover, all trained models are evaluated on a fully unseen, real-world OTA dataset collected across 13 distinct positions, enabling the assessment of the model’s generalization performance.

## 3. Dataset Generation

Based on the 2D amplitude-phase histogram representation introduced in ref. [5], five different datasets, comprising both non-HT and HT IEEE 802.11 OFDM signals, were generated to assess the performance of the proposed CNN architecture in classifying OFDM modulations under multipath fading and noise conditions. Following the training phase, the models are tested in OTA scenarios. The generation methodology for both training and testing datasets is detailed in the subsequent section.

### 3.1. Generation of Training Datasets

This work analyses the effects of multipath fading, noise, and synchronization impairments (i.e., symbol clock and carrier frequency) within the training data. Furthermore, it examines the impact of hardware-specific equipment signatures on the captured signals. To this end, three distinct signal impairment conditions are considered: (1) synthetic data featuring multipath fading and noise while assuming perfect symbol and carrier synchronization; (2) synthetic data incorporating multipath fading, noise, and synchronization errors; and (3) conducted (cable-based) data acquired through direct transmission, capturing real hardware impairments in addition to multipath fading and noise.

The resulting datasets (i.e., (D1) mid-scale perfectly synchronized synthetic dataset, (D2) mid-scale synchronization-impaired synthetic dataset, (D3) large-scale synchronization-impaired synthetic dataset, (D4) small-scale synchronization-impaired synthetic dataset, and (D5) small-scale conducted dataset) represent a trade-off between data scale and hardware realism: the simulated datasets enable large-scale training and controlled impairment modeling, whereas the conducted dataset, although smaller due to practical data acquisition constraints, captures authentic and unmodeled RF distortions introduced by real hardware. This design facilitates a simulation-to-reality analysis of whether models trained on large volumes of simulated variability can generalize effectively to unseen OTA channel conditions compared to models trained on a smaller, but physically realistic hardware dataset.

#### 3.1.1. Synthetic Data Without Synchronization Impairments

To generate the 2D amplitude-phase histograms, 20 MHz IEEE 802.11 signals with 52 data subcarriers were simulated in MATLAB R2025b [20]. First, 8-symbol data fields were generated for BPSK, QPSK, 16QAM, and 64QAM modulations (corresponding to modulation and coding schemes (MCSs) 0, 1, 3, and 5). The resulting signals were passed through indoor TGn multipath fading models (types A–F) [21], compensated for filter delays, and demodulated, with the 16-sample cyclic prefix removed.

To emulate realistic reception conditions, measured AWGN was added over an SNR range from 0 to 36 dB in 3 dB increments. The noisy QAM symbols were then grouped into blocks of 128 OFDM symbols, while amplitude outliers exceeding the 99th percentile were discarded.

**Figure 1:**
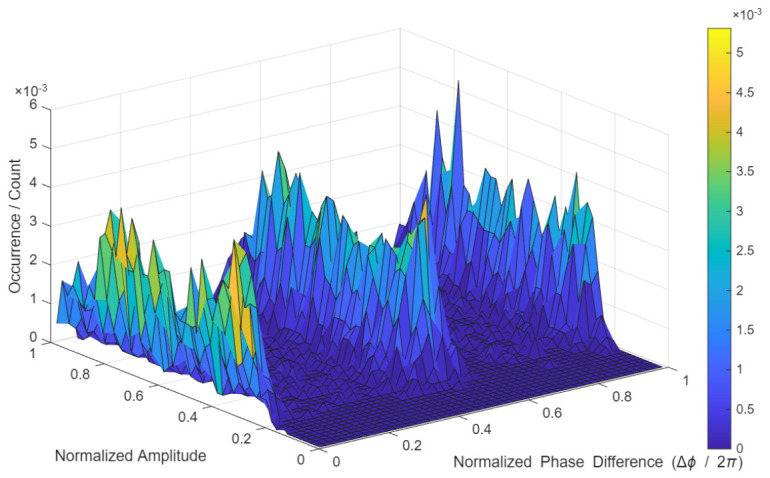
2D amplitude-phase histogram representation of BPSK signal.

Using only the data subcarriers, the amplitudes were normalized by the maximum value within the 99% interval, while the phase difference between consecutive symbols was normalized by 2π. These amplitude-phase pairs were mapped onto 2D histograms over a 1 × 1 grid with a resolution of 1/50 per axis, as illustrated in Figure 1.

This process generates 1562 histograms for each combination of MCS, SNR, and channel model, resulting in a total of 487,344 histograms. The resulting dataset represents the perfectly synchronized dataset D1.

#### 3.1.2. Synthetic Data with Synchronization Impairments

**Figure 2:**
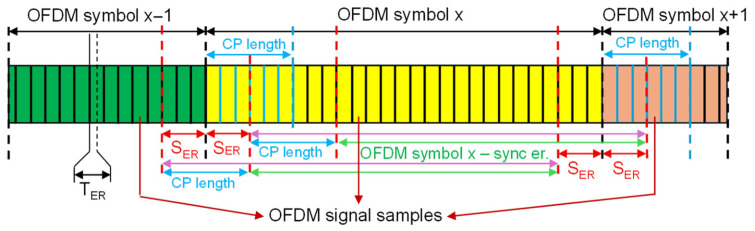
OFDM demodulation window under symbol clock synchronization errors.

**Figure 3:**
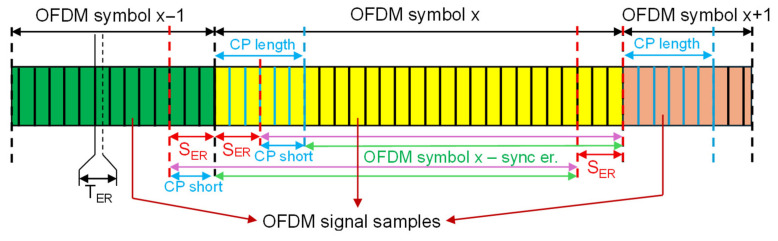
Mitigation of inter-symbol interference via reduced cyclic prefix.

To create comprehensive datasets, signals affected by symbol clock and carrier synchronization impairments were generated and combined with the previously described ideal-synchronization data. These impairments were injected prior to the OFDM demodulation step to accurately reflect real-world receiver inaccuracies. Figure 2 and Figure 3 present the timing uncertainties introduced during the generation process.

First, sampling and frequency inaccuracies are introduced. Fractional sampling errors $T_{ER}$ are simulated by shifting the actual sampling moment from its ideal position by a random fraction of the sampling period, utilizing a fine precision step of $T_{ER}$/1000. Alongside this, a carrier frequency offset (CFO) is applied relative to the subcarrier frequency spacing. This frequency shift introduces a dynamic, cumulative phase rotation across the samples of successive OFDM symbols.

Second, symbol clock synchronization errors $S_{ER}$ are injected to emulate timing uncertainty, utilizing two distinct strategies to handle the demodulation window. In the first approach, illustrated in Figure 2, a timing offset is applied where the advanced starting sample simply reduces the effective guard interval, denoted by CP length. Conversely, a delayed start utilizes the full guard interval but inevitably causes samples to be erroneously read from the subsequent OFDM symbol. The second approach, presented in Figure 3, attempts to mitigate the inter-symbol interference by artificially decreasing the length of the considered cyclic prefix (i.e., CP short). By deliberately reducing the demodulation window, it ensures that even with a delayed start, all samples extracted for demodulation belong to the current symbol.

To comprehensively emulate the synchronization errors, three distinct impairment scenarios were configured, combining specific values for timing errors, frequency deviations, and fractional sampling shifts. To construct the expanded dataset, these scenarios were systematically combined with the base signal parameters. Specifically, for every unique combination of modulation scheme, multipath channel model, and SNR level, the base signals were replicated and independently subjected to each of the three impairment configurations.

The configurations are summarized in Table 1.

Following the insertion of these specific timing and frequency impairments, the signals continue the previously described processing pipeline, undergoing the same demodulation, noise addition, normalization, and two-dimensional histogram generation steps to produce the final, expanded dataset. The resulting dataset (D3) comprises 1,947,117 histograms, including both impairment-free and synchronization-impaired data, thereby providing the diversity required for training a DL model.

To systematically investigate the impact of dataset scale and synthetic augmentation on model performance, two additional subsets (D2 and D4) are derived from the large-scale synchronization-impaired dataset. These subsets are constructed to preserve a uniform distribution with respect to modulation scheme, signal-to-noise ratio (SNR), and channel model. The first subset (D2) consists of 487,344 histograms, matching the size of the perfectly synchronized dataset; this enables an assessment of the effect of incorporating synchronization errors into the training data. The second subset (D4) comprises 18,591 histograms, corresponding in size to the conducted hardware dataset, thereby facilitating a direct comparison between models trained on synthetic and hardware-transmitted data. Furthermore, by training the proposed CNN on datasets of three distinct sizes, the influence of dataset scale on model performance can also be evaluated.

#### 3.1.3. Conducted Data with Hardware-Specific Impairments

To study the effect of real hardware imperfections (i.e., DC offsets, IQ imbalance, phase noise, quantization errors, local oscillator drift, and power amplifier nonlinearities) on model training, the last dataset (D5) was generated using SDR platforms. Two hardware configurations were used to generate the data: (1) a local loopback on a single BladeRF x40 board [22] (directly connecting its own RF output to its input), and (2) a two-board setup where one SDR acted as the dedicated transmitter and the other as the receiver. This controlled approach introduces authentic radio interface impairments while maintaining strict control over the channel characteristics. Because the coaxial cable itself contributes minimal noise and fading, the 20 MHz IEEE 802.11 radio frames were digitally pre-filtered to simulate TGn multipath channels (A–F, plus an ideal channel) and subjected to additive noise (ranging from 0 to 30 dB in 2 dB steps) prior to transmission.

The transmitted signal was upsampled to a 25 MHz sampling frequency. To ensure a balanced dataset, two MCSs were assigned to each core modulation type. Transmissions were grouped into batches of 25,000 frames, with data organized into random blocks of 2 consecutive OFDM bursts sharing the same MCS, channel model, and SNR. To bypass the need for complex IEEE 802.11 header demodulation at the receiver, custom signalling sequences were prepended to the data bursts. These 512-sample sequences utilized a 2-of-6 multifrequency code (using frequencies between 1.25 MHz and 7.5 MHz) to encode the MCS, the channel model, and the SNR, as presented in Table 2.

At the receiver, the incoming 25 MHz signal first passed through a high-pass DC blocker to mitigate hardware-induced imbalances before being downsampled back to the standard 20 MHz. It should be noted that the cabled connection between the transmitter and receiver introduced variable DC offsets, which the DC-blocking stage could only partially suppress.

**Figure 4:**
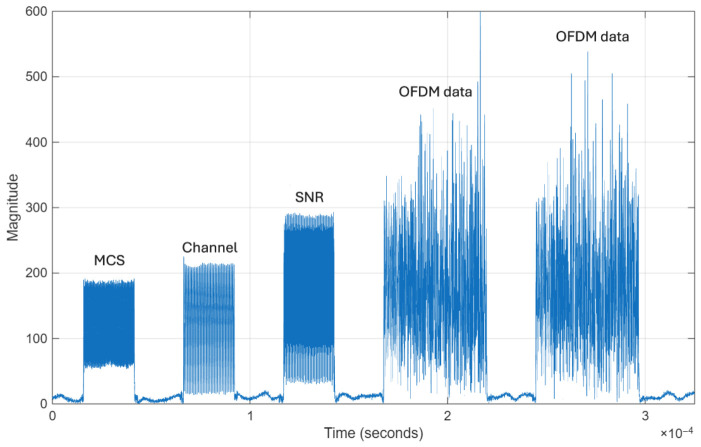
Time-domain representation of the received signal frame.

Figure 4 shows the time-domain amplitude of the received signal, capturing a transmission sequence that reflects the system’s frame structure. Each sequence begins with a custom preamble consisting of three narrow, 512-sample multifrequency signaling bursts—used to decode transmission parameters such as the MCS, channel model, and SNR—followed by two OFDM data bursts.

To accurately isolate the signalling sequences and data frames, a burst detection algorithm was applied. This involved processing the absolute value of the signal through a dual moving-average smoothing filter, followed by a hysteresis comparator. If a detected burst failed to meet strict length conditions—approximately 512 samples for signalling and 1500 for data—the entire group was discarded. The remaining signalling sequences then underwent spectral analysis to decode the ground-truth parameters, and any sequence yielding an invalid frequency combination triggered the rejection of that frame group.

Once isolated and validated, the data frames underwent the hardware synchronization pipeline. This included symbol timing estimation, followed by both coarse and fine carrier frequency offset estimation and correction. After stripping the frame header, 8 OFDM payload symbols were retained and fed directly into the previously established processing pipeline. They were demodulated, organized into groups of 128 OFDM symbols, normalized, and mapped into two-dimensional histograms just as in the simulated datasets. This batch-processing workflow generated a final dataset (D5) of 18,591 histograms. It should be noted that the conducted dataset (~18,500 samples) is representative of typical real-world measurement conditions, where large-scale acquisition is constrained by acquisition time, storage requirements, and environmental variability during extended recording sessions.

### 3.2. Generation of OTA Testing Datasets

To assess the models’ robustness against complex real-world RF effects, including transceiver nonlinearities and ambient interference, an OTA wireless dataset was collected in a physical indoor environment. This testing phase utilized two BladeRF x40 platforms, communicating over a wireless link. Unlike the conducted cable tests, no synthetic noise or digital channel pre-filtering was applied to the IEEE 802.11 frames. Instead, the upsampled transmissions were subjected entirely to the natural multipath fading, spatial interference, and noise of the real-world physical environment, alongside the inherent hardware impairments of the radio interfaces.

To ensure the transmission algorithm maintained a consistent frame format across all experiments, the same three multifrequency signalling sequences were prepended to the data bursts. However, because the channel and noise were natural rather than simulated, only the first sequence actively encoded variable ground-truth information, namely the selected MCS.

Upon reception, the signals passed through the previously established hardware processing pipeline, obtaining the 2D histograms. Because the physical environment determines signal degradation, the final testing dataset is categorized exclusively by the intended MCS and the true receiver-estimated SNR, calculated from the IEEE 802.11 Long Training Field (LTF). This results in a robust set of histograms that accurately reflects real OTA reception conditions.

The OTA testing datasets were generated by conducting wireless transmissions in various indoor settings within the Technical University of Cluj-Napoca. Four distinct testing locations were utilized to capture different multipath and fading characteristics: (1) the entrance hall from the ground floor, (2) the hall area from the 5th floor, (3) the corridor from the 5th floor, and (4) a laboratory room (room 510). Within each environment, the spatial separation between the transmitter and receiver SDR platforms was systematically adjusted to establish 13 distinct testing positions.

**Figure 5:**
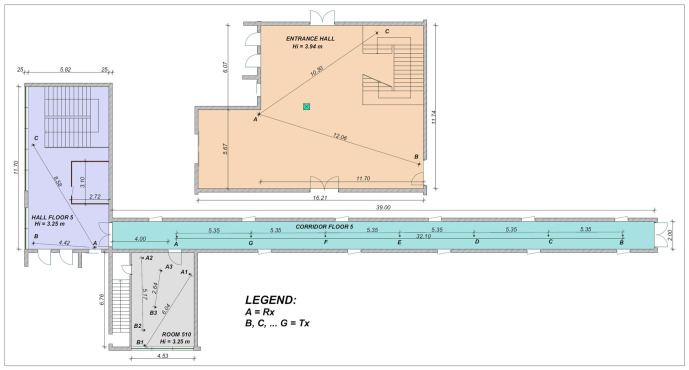
Floor plans and measurement positions.

Figure 5 illustrates the floor plans of the selected environments, detailing the exact measurement coordinates. In each layout, point A denotes the receiver’s location, whereas points B through G indicate the corresponding transmitter positions.

The total number of histograms collected at each position is summarized in Table 3.

**Figure 6:**
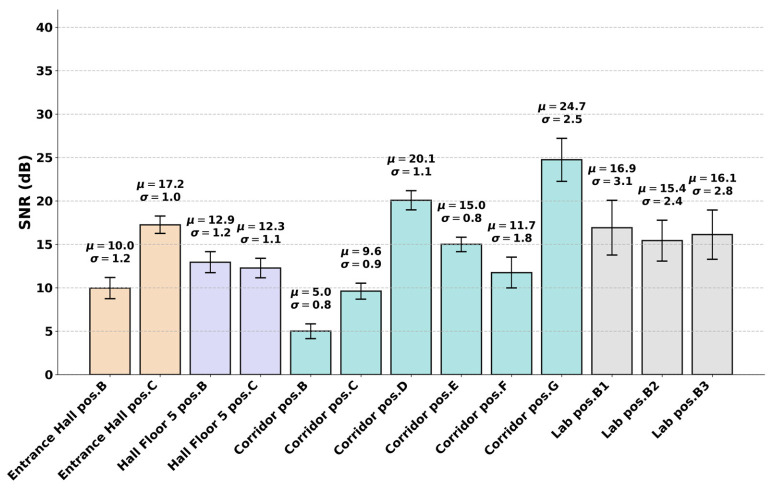
SNR quality across OTA scenarios.

By changing the testing positions, the signals were subjected to different degrees of attenuation, reflections, and spatial interference, resulting in a diverse range of received SNRs for each specific position. Figure 6 describes the detailed SNR quality, represented by the mean μ and standard deviation σ, measured across each scenario.

As illustrated in the chart, the measured SNR varied significantly depending on the environment and the distance between the transceivers, enabling a more comprehensive evaluation of the models under various propagation conditions.

**Figure 7:**
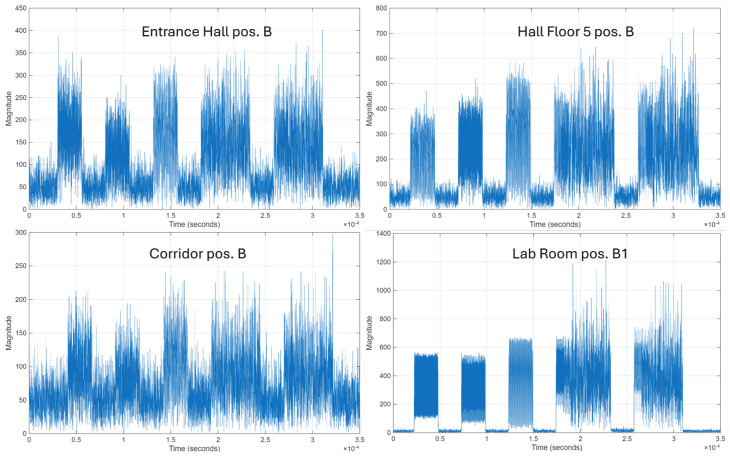
Time-domain representation of the received signal frames in distinct measurement conditions.

Figure 7 presents the time-domain representation of the received signal frames in different measurement conditions.

These plots visually confirm the varying levels of path loss and multipath fading introduced by the indoor channel. Across all scenarios, the received signals manifest noticeable attenuation, with the severity depending on the distance between the transmitter and receiver, as well as the specific reflection characteristics of the room.

Compared to the scenario in which the lab room is positioned at B1, which presents the highest signal magnitude, greater transmission distance and increased environmental complexity result in more pronounced multipath fading, characterized by significant fluctuations in the signal envelope. The most severe degradation is observed in the corridor position B scenario, where path loss and multipath fading drastically reduce the signal amplitude, allowing the noise floor to partially mask the packet boundaries.

## 4. Model Training

Following their generation, the previously described datasets serve as the foundation for training and evaluating a CNN architecture dedicated to modulation recognition.

### 4.1. Model Architecture

**Figure 8:**
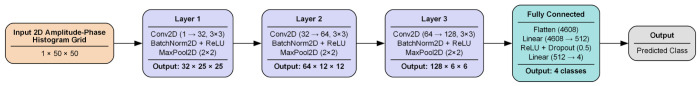
CNN architecture.

The proposed CNN architecture, presented in Figure 8, is a deep hierarchical feature extractor designed to classify signal modulations by processing the spatial density patterns within 2D amplitude-phase histograms.

Drawing inspiration from the VGG [23] architectural paradigm, the proposed CNN is designed to process an input tensor of size 1 × 50 × 50, representing the 2D amplitude-phase histogram grid. The network features three sequential convolutional blocks utilizing compact 3 × 3 kernels to capture local geometric symmetries and constellation clusters inherent in the histogram representation.

As the network deepens, the number of convolutional filters progressively doubles (from 32 to 64, and finally to 128), extracting increasingly complex hierarchical features. Each convolution is integrated with Batch Normalization to stabilize training and a ReLU activation function to model non-linear statistical distributions. Systematic spatial downsampling is achieved via 2 × 2 Max-Pooling layers at the end of each block, reducing the spatial resolution from 50 × 50 down to 6 × 6.

The architecture then transitions into a fully connected classification head that flattens the resulting 128 × 6 × 6 feature map (4608 elements) into a 512-dimensional hidden layer. To ensure robust generalization, a dropout rate of 0.5 is applied prior to the final linear layer, which maps the high-level abstractions to the 4 target modulation schemes.

### 4.2. Training Hyperparameters

The datasets are divided into training and validation sets using an 80/20 split. To emphasize the structural topology and geometric signatures of the modulation schemes, rather than absolute sample intensities, each 2D histogram is independently normalized to the [0, 1] interval via per-instance maximum normalization.

Model training is performed using the Adaptive Moment Estimation (Adam) optimizer and Cross-Entropy loss function, with a batch size of 1024, a learning rate of $10^{-3}$ and a weight decay of $10^{-4}$. An early stopping mechanism with a 10-epoch patience window is used to stop training once validation loss stabilizes, ensuring the selection of the best-performing weights.

The training is performed using the PyTorch library [24] (https://pytorch.org/ (accessed on 26 March 2026)) on an NVIDIA A100-PCIE GPU [25] with 40 GB of RAM. To ensure statistical reliability and account for random weight initialization, the model is trained 10 times independently for each experimental case. This multi-trial evaluation strategy is consistent with established deep learning methodologies, which emphasize that statistical validation is essential to mitigate the random nature of network convergence [26,27].

### 4.3. Computational Complexity

Regarding computational complexity, the network contains 2,454,980 trainable parameters with a total memory footprint of approximately 9.37 MB, requiring 25.77 million Multiply–Accumulate operations per forward pass. Baseline latency testing on a standard commercial workstation equipped with an NVIDIA GeForce RTX 5070 GPU yielded an average execution time of 0.374 ms per sample, demonstrating that the architectural complexity is sufficiently low to process inferences rapidly without creating processing bottlenecks on embedded hardware.

## 5. Results

To systematically isolate the effects of dataset scale, synthetic impairments, and hardware-induced signal impairments on cross-domain generalization, the CNN architecture is evaluated under five distinct training scenarios: (D1) a mid-scale perfectly synchronized synthetic dataset with AWGN and multipath fading effects, (D2) a mid-scale synchronization-impaired synthetic dataset (i.e., AWGN, multipath fading effects, and synchronization errors), (D3) a large-scale synchronization-impaired synthetic dataset, (D4) a small-scale synchronization-impaired synthetic dataset, and (D5) a small-scale hardware conducted dataset.

The subsequent evaluation is structured into two primary phases. In the first phase, the five training strategies are compared through three key analyses: (1) their overall mean OTA accuracy alongside their associated standard deviation and 95% confidence interval, (2) their per-class classification performance, and (3) their accuracy as a function of the estimated SNR.

In the second phase, a detailed statistical analysis is conducted on the best-performing model. Its classification performance and robustness to spatial fading are assessed using: (1) confusion matrix, (2) class-wise accuracy across varying SNR levels, and (3) generalization consistency across different physical test positions.

To ensure statistical reliability, all reported metrics are averaged over the ten independent training runs.

### 5.1. Cross-Domain Generalization Performance

The initial phase of the evaluation systematically benchmarks the five training strategies against real-world indoor OTA signals. The results of this comparative analysis, summarized in Table 4, present a comprehensive overview of the overall classification performance, revealing several key insights into the models’ cross-domain generalization.

Comparing the two mid-scale datasets, the inclusion of synchronization errors leads to a modest improvement: the model trained on D2 achieves a slightly higher mean accuracy (92.20%) than the baseline D1 (91.54%). Moreover, their 95% interval do not overlap, suggesting that modeling symbol clock and carrier frequency offsets during training can help better align the learned decision boundaries with OTA distortions.

The impact of dataset scale is also observable. Model D3, trained on the large-scale synchronization-impaired dataset, attains the highest mean accuracy (93.36%) along with a relatively low standard deviation (±0.42%). This indicates that increased exposure to simulated variability may contribute to improved robustness and stable performance.

A direct comparison can be made between the volume-matched small-scale datasets: the small-scale synchronization-impaired dataset (D4) and the small-scale conducted hardware dataset one (D5). Their comparable mean accuracies (89.89% vs. 89.38%) indicate that, at this restricted scale, algorithmically generated impairments provide equivalent distributional diversity for OTA generalization as physical RF captures. Additionally, both small-scale models exhibit the highest variability, with standard deviations of ±0.52% and ±0.60%. This indicates that limited training volume tends to reduce generalization stability, regardless of whether the data source is synthetic or physical.

**Figure 9:**
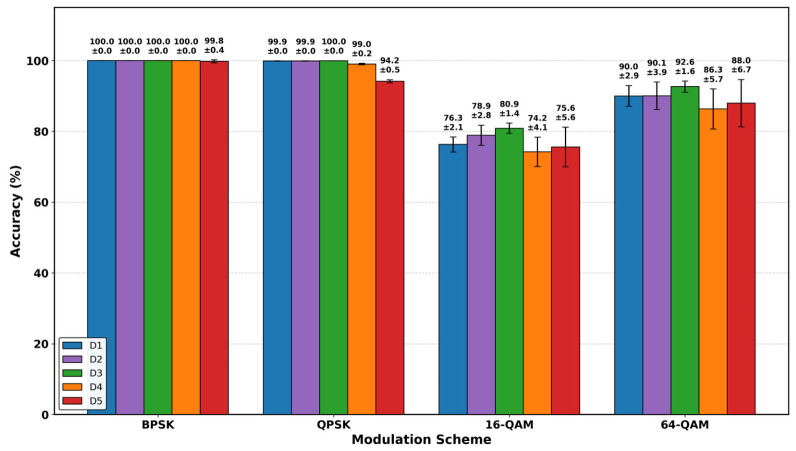
Classification accuracy per modulation scheme across training datasets (D1–D5).

While these overall metrics highlight general training stability, the per-class classification results, presented in Figure 9, provide a more detailed view of the models’ generalization behavior across modulation formats.

For lower-order schemes such as BPSK and QPSK, all models achieve near-perfect performance, indicating that sparse constellation structures are relatively robust to the domain shift between training and OTA conditions. In contrast, performance differences become more evident for higher-order modulations, particularly 16-QAM and 64-QAM, where denser constellation points require more precise decision boundaries and are more sensitive to channel distortions.

Among the evaluated models, dataset D3, trained on the large-scale synchronization-impaired synthetic data, consistently achieves the strongest performance on these higher-order modulation schemes, with peak accuracies of 80.9% for 16-QAM and 92.6% for 64-QAM, while also exhibiting comparatively low variability. Consistent with earlier observations, this suggests that scaling dataset volume is essential for learning highly robust decision boundaries in higher-complexity regimes.

Comparing the mid-scale models further indicates a modest advantage for the inclusion of synchronization errors. Model D2 slightly outperforms the baseline D1 on 16-QAM (78.9% vs. 76.3%), suggesting that explicitly modeling synchronization impairments can improve robustness to real-world distortions.

Furthermore, the small-scale models (D4 and D5) exhibit lower accuracy and higher variance, particularly for higher-order modulations. This reflects reduced stability in learned decision boundaries under limited training data, making performance more sensitive to stochastic channel effects, regardless of whether the data is synthetically generated or collected from hardware.

**Figure 10:**
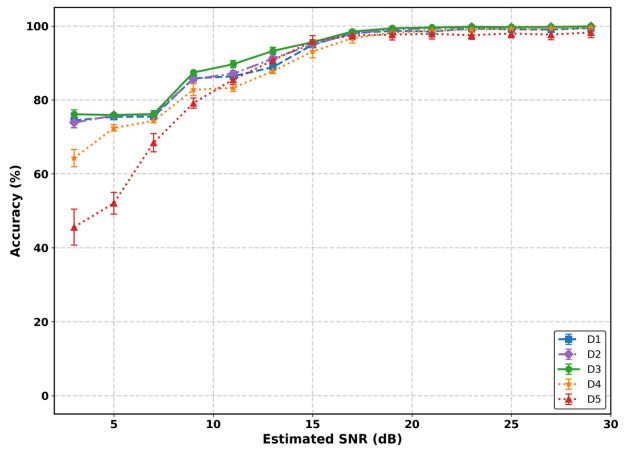
Classification accuracy vs. estimated SNR across training datasets (D1–D5).

Building on these observations, Figure 10 explores model robustness under realistic channel variability by detailing classification accuracy as a function of the estimated SNR, evaluated across discrete 2 dB intervals.

In the high-SNR regime (above 20 dB), models trained on synthetic datasets (D1–D4) converge toward near-saturation performance, indicating that all synthetic training strategies effectively capture the underlying modulation structure when channel noise is minimal. The hardware-trained model (D5) reaches a marginally lower performance ceiling, though the difference is negligible.

The most pronounced differences emerge in the low-SNR regime (below 10 dB), where classifier robustness is most severely challenged. Here, model D3 consistently achieves the highest accuracy across all noise levels, demonstrating once again that dataset scale and diversity play a dominant role in enhancing noise resilience.

The impact of cable-induced impairments becomes particularly evident when comparing the volume-matched, small-scale datasets. At low SNR, the large-scale synchronization-impaired synthetic dataset (D4) exhibits the expected performance degradation, whereas the conducted hardware dataset (D5) declines more sharply, falling below 50% accuracy at the lowest SNR levels. The increasing variance in D5’s performance—evidenced by widening error bars—further highlights its limited statistical coverage under noisy conditions. This reduced robustness in low-SNR regimes is likely driven by hardware-specific artifacts, such as DC offsets introduced by the cabled acquisition setup. Because these specific anomalies are absent in the OTA test environment, they induced a distributional shift that limits the model’s generalization capabilities, particularly when attempting to resolve complex synchronization errors induced by real-world multipath fading.

Overall, these results show that dataset scale and diversity play a more decisive role than data provenance alone in achieving robust generalization. While data derived from conducted measurements introduces realistic hardware impairments, it can also embed hardware-specific artifacts that bias the learned representations. In contrast, large-scale datasets with varied synthetic synchronization impairments provide broader and more controlled coverage of channel conditions, effectively regularizing the model and improving robustness under severely degraded environments.

### 5.2. Class Robustness and Spatial Fading Analysis

In the second phase, the best-performing model—trained on the D3 dataset consisting of large-scale synchronization-impaired data—is selected for a more detailed evaluation of classification performance and robustness to spatial fading.

**Figure 11:**
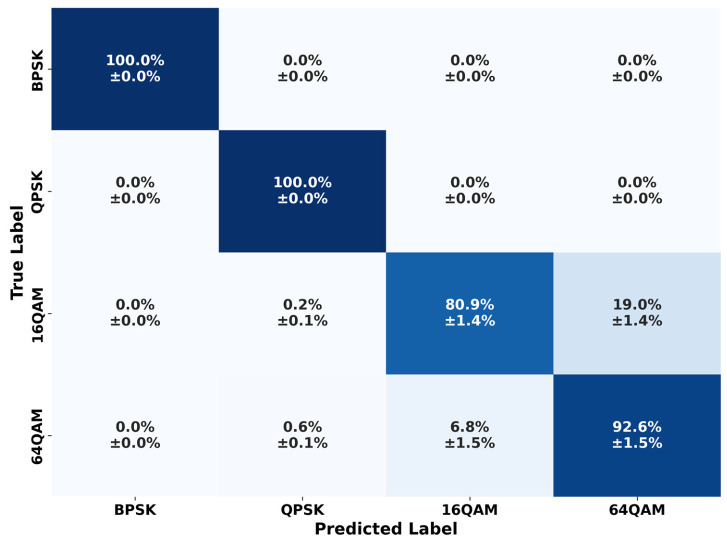
Statistical confusion matrix.

The analysis begins with the statistical confusion matrix, presented in Figure 11.

The confusion matrix highlights high classification fidelity with near-perfect performance (100%) for BPSK and QPSK modulations. The primary source of error is an asymmetric confusion between the QAM schemes, where 19.0% of 16-QAM signals are misclassified as 64-QAM. This suggests that real-world distortions cause the 16-QAM constellation to spread into the denser 64-QAM decision space. Nevertheless, the model maintains a robust 92.6% accuracy for 64-QAM with low statistical variance (±1.5%), confirming that the massive-scale training successfully established stable decision boundaries for high-order modulations.

**Figure 12:**
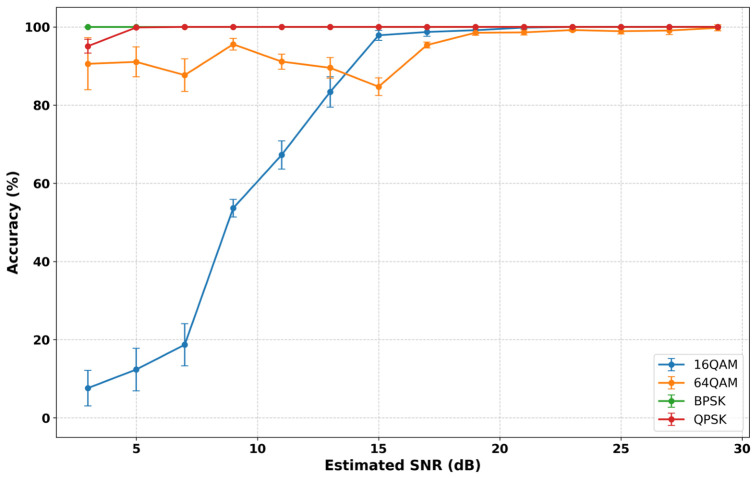
Per-class mean accuracy vs. estimated SNR.

A more detailed explanation of these QAM misclassifications is provided in the per-class SNR analysis shown in Figure 12, which illustrates classification accuracy as a function of the estimated SNR evaluated across discrete 2 dB intervals.

While BPSK and QPSK remain perfectly resilient across the entire power spectrum, the performance of the QAM schemes is heavily dependent on the noise floor.

Specifically, 16-QAM exhibits a sharp degradation below 15 dB, which directly explains the misclassification rate seen in the confusion matrix. As the SNR decreases, the 16-QAM constellation points become indistinct, leading the model to misclassify them as the denser 64-QAM scheme. However, 64-QAM maintains a more stable performance at lower SNRs compared to 16-QAM, though it carries high statistical variance. Both high-order modulations eventually converge to 100% accuracy above 20 dB, confirming that the model’s primary classification challenges are confined to high-noise regimes where constellation points overlap.

**Figure 13:**
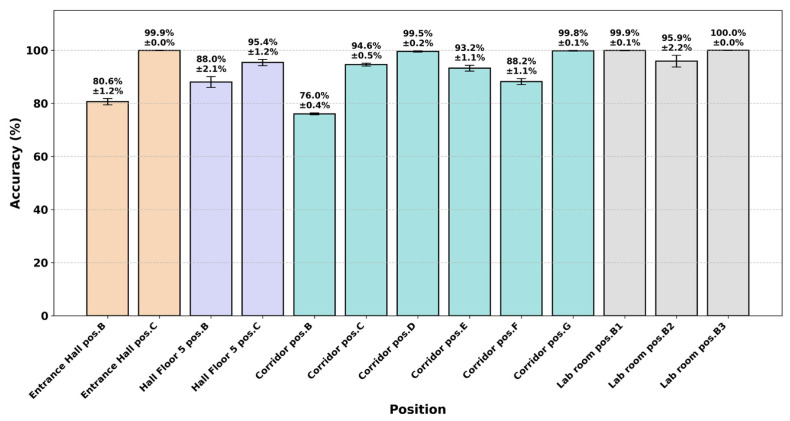
Mean accuracy across testing positions.

Because the challenging conditions are linked to the physical testing environment, evaluating the spatial robustness of training scenario D3 across the 13 indoor positions reveals a direct correlation between local SNR quality and classification consistency. Figure 13 details the mean classification accuracy achieved across all 13 testing positions.

In high-SNR environments such as corridor position G (24.7 dB) and lab room position B3 (16.1 dB), the model maintains near-perfect accuracy (up to 100.0%), proving that its learned features are invariant to spatial shifts in clear signal conditions.

In contrast, performance decreases in low-SNR environments. At corridor position B (5.0 dB), the accuracy drops to 76.0%, which is the lowest observed value. This matches earlier findings that higher-order QAM schemes are more affected by noise below 10 dB.

Despite these variations, the model demonstrates remarkable stability, with low standard deviations (typically ≤ 1.2%) even in challenging environments. This confirms that the combination of large-scale training data and synthetic impairments helps the model generalize well across different indoor fading scenarios and reduces sensitivity to spatial changes.

It should be mentioned that although the current OTA evaluation is conducted in a static indoor environment, the proposed signal representation is inherently structured to support dynamic, outdoor scenarios affected by high mobility and Doppler shifts. Specifically, the 128-symbol amplitude-phase histograms are constructed by aggregating 16 independent groups of 8 OFDM symbols. In a highly mobile scenario—such as vehicular speeds of 100 km/h—the channel coherence time is approximately 2 ms in the 2.4 GHz band and 1 ms in the 5.8 GHz band. Because the duration of an 8-symbol group in a 20 MHz bandwidth is only 32 μs, it is much smaller than the coherence time. Consequently, the channel transfer function remains effectively constant within each 8-symbol block, but varies across the 16 blocks that make up a single histogram. This block-wise data organization ensures that the histogram naturally captures the statistical distribution of time-selective fading without suffering from intra-block distortion, indicating that the architecture would remain highly robust in dynamic mobility scenarios.

However, the scope of these conclusions is strictly bounded to controlled indoor environments, and we do not attempt to generalize these results to outdoor or high-mobility applications.

## 6. Conclusions

This paper evaluated the cross-domain generalization capability of a CNN architecture in classifying digital modulation schemes (BPSK, QPSK, 16-QAM, and 64-QAM) under realistic indoor OTA channel conditions. To systematically isolate the effects of dataset scale, synthetic impairments, and hardware-induced signal impairments, the architecture’s classification performance was benchmarked across five distinct training scenarios: (D1) a mid-scale perfectly synchronized synthetic dataset, (D2) a mid-scale synchronization-impaired synthetic dataset, (D3) a large-scale synchronization-impaired synthetic dataset, (D4) a small-scale synchronization-impaired synthetic dataset, and (D5) a small-scale conducted hardware dataset. To ensure statistical reliability, each scenario was independently trained ten times. The trained models were subsequently tested against an unseen OTA dataset captured across 13 diverse indoor spatial positions to explicitly quantify the simulation-to-reality gap.

First, the results show that combining a large dataset scale with synthetic synchronization impairments significantly improves cross-domain robustness. This approach provides the training diversity needed to stabilize decision boundaries, achieving the highest overall OTA accuracy (93.36%). Large datasets proved especially beneficial for accurately classifying higher-order modulations (such as 16-QAM and 64-QAM) and maintaining steady performance in moderate-to-low SNR environments.

Second, the evaluation highlights the clear limitations of restricted training data. Both small-scale models—whether synthetic or hardware-based—exhibited lower accuracies and higher variance, indicating that limited training data reduces overall performance stability. Furthermore, using real conducted measurements introduces a domain shift due to cabled artifacts (e.g., DC offsets). Because these specific artifacts are not present in pure OTA conditions, they limit the model’s generalization capabilities under low-SNR conditions.

Following the demonstration of the model’s generalization capability through the independent evaluation of synthetic and hardware data sources, future research will investigate hybrid training strategies to further improve classification performance. A particularly promising direction is the use of transfer learning, where the network is initially pre-trained on large, augmented synthetic datasets to learn general decision boundaries, and then fine-tuned using limited OTA hardware measurements to capture transceiver-specific nonlinearities. Additionally, extending the evaluation to include mobile receivers and dynamic, time-varying channels will enable a more comprehensive assessment of the architecture’s robustness in realistic wireless network conditions.

## Figures and Tables

**Table 1:** Configuration of impairment scenarios.

Demodulation Window	Timing Error (*S_ER_*)	Frequency Deviation (CFO)	Maximum Fractional Sampling Error
Standard (Full Length)	2 samples	5%	1 × $T_{ER}$
Reduced (CP short)	4 samples	5%	1 × $T_{ER}$
Reduced (CP short)	2 samples	0.25%	0.2 × $T_{ER}$

**Table 2:** Multifrequency code allocation scheme.

Frequency Values (MHz)	Encoded MCS	Modulation	Encoded Channel Model	Encoded SNR (dB)
1.25 + 2.5	-	-	-	0–2
1.25 + 3.75	MCS 0	BPSK	-	2–4
1.25 + 5.0	MCS 1	BPSK	Ideal	4–6
1.25 + 6.25	MCS 2	QPSK	Model A	6–8
1.25 + 7.5	MCS 3	QPSK	Model B	8–10
2.5 + 3.75	MCS 4	16QAM	Model C	10–12
2.5 + 5.0	MCS 5	16QAM	Model D	12–14
2.5 + 6.25	MCS 6	64QAM	Model E	14–16
2.5 + 7.5	MCS 7	64QAM	Model F	16–18
3.75 + 5.0	-	-	-	18–20
3.75 + 6.25	-	-	-	20–22
3.75 + 7.5	-	-	-	22–24
5.0 + 6.25	-	-	-	24–26
5.0 + 7.5	-	-	-	26–28
6.25 + 7.5	-	-	-	28–30

**Table 3:** Number of histograms for each testing position.

Testing Position	No. of Histograms
Entrance Hall pos. B	3270
Entrance Hall pos. C	3271
Hall Floor 5 pos. B	3268
Hall Floor 5 pos. C	3190
Corridor pos. B	2738
Corridor pos. C	3270
Corridor pos. D	3271
Corridor pos. E	3270
Corridor pos. F	3259
Corridor pos. G	3260
Lab room pos. B1	3254
Lab room pos. B2	3264
Lab room pos. B3	3259

**Table 4:** Overview of training datasets and OTA classification performance.

Index	Training Dataset	Training Size	Impairment Conditions	OTA Accuracy (μ±σ)	95% Confidence Interval
D1	Mid-scale perfectly synchronized synthetic dataset	487,344	Multipath fading + AWGN	91.54%±0.29%	[91.33%, 91.75%]
D2	Mid-scale synchronization-impaired synthetic dataset	487,344	Multipath fading + AWGN + Sync. errors	92.20%±0.54%	[91.81%, 92.56%]
D3	Large-scale synchronization-impaired dataset	1,947,117	Multipath fading + AWGN + Sync. errors	93.36%±0.42%	[93.06%, 93.66%]
D4	Small-scale synchronization-impaired dataset	18,591	Multipath fading + AWGN + Sync. errors	89.89%±0.52%	[89.52%, 90.26%]
D5	Small-scale hardware dataset	18,591	Multipath fading + AWGN + Conducted RF hardware impairments	89.38%±0.60%	[88.95%, 89.81%]

## Data Availability

The generated 2D amplitude-phase histogram datasets supporting the findings of this study are openly available on Hugging Face at [6].

## Abbreviations

The following abbreviations are used in this manuscript:

RF	Radio Frequency
AMC	Automatic Modulation Classification
OFDM	Orthogonal Frequency Division Multiplexing
CNN	Convolutional Neural Network
OTA	Over-the-Air
IoT	Internet of Things
CR	Cognitive Radio
SDR	Software-Defined Radio
DL	Deep Learning
I/Q	In-phase and Quadrature
HT	High Throughput
VHT	Very High Throughput
HE	High Efficiency
CFO	Carrier Frequency Offset
AWGN	Additive White Gaussian Noise
SNR	Signal-to-Noise Ratio
MCS	Modulation and Coding Scheme
LTF	Long Training Field
GPU	Graphics Processing Unit
BPSK	Binary Phase Shift Keying
QPSK	Quadrature Phase Shift Keying
QAM	Quadrature Amplitude Modulation
